# Effect of Ouabain on Glutamate Transport in the Hippocampus of Rats with LPS-Induced Neuroinflammation

**DOI:** 10.3390/biomedicines11030920

**Published:** 2023-03-16

**Authors:** Israel José Pereira Garcia, Paula Fernanda Kinoshita, Jéssica Martins de Moura Valadares, Luciana Estefani Drumond de Carvalho, Vanessa Faria Cortes, Leandro Augusto Barbosa, Cristoforo Scavone, Hérica de Lima Santos

**Affiliations:** 1 Cellular Biochemistry Laboratory, Federal University of São João del-Rei, Campus Cento-Oeste, Divinópolis 35501-296, Brazil; 2 Membrane and ATPase Biochemistry Laboratory, Federal University of São João del-Rei, Campus Cento-Oeste, Divinópolis 35501-296, Brazil; 3 Molecular Neuropharmacology Laboratory, Department of Pharmacology, Institute of Biomedical Science, University of São Paulo, São Paulo 05508-000, Brazil

**Keywords:** ouabain, LPS, EAATs, Na, K-ATPase, FXYD2, glutamate, hippocampus

## Abstract

A lipopolysaccharide (LPS)-induced neuroinflammation rat model was used to study the effects of ouabain (OUA) at low concentrations, which can interact with the Na,K-ATPase, causing the modulation of intracellular signalling pathways in the Central Nervous System. Our study aimed to analyse the effects of OUA on glutamate transport in the hippocampus of rats with LPS-induced neuroinflammation. Adult male Wistar rats were divided into four groups: OUA (1.8 µg/kg), saline (CTR), LPS (200 µg/kg), and OUA + LPS (OUA 20 min before LPS). The animals were sacrificed after 2 h, and the hippocampus was collected for analysis. After treatment, we determined the activities of Na,K-ATPase and glutamine synthetase (GS). In addition, expression of the α1, α2, and α3 isoforms of Na,K-ATPase and the glutamate transporters, EAAT1 and EAAT2, were also analysed. Treatment with OUA caused a specific increase in the α2 isoform expression (~20%), whereas LPS decreased its expression (~22%), and treatment with OUA before LPS prevented the effects of LPS. Moreover, LPS caused a decrease of approximately 50% in GS activity compared with that in the CTR group; however, OUA pre-treatment attenuated this effect of LPS. Notably, it was found that treatment with OUA caused an increase in the expression of EAAT1 (~30%) and EAAT2 (~25%), whereas LPS caused a decrease in the expression of EAAT1 (~23%) and EAAT2 (~25%) compared with that in the CTR group. When treated with OUA, the effects of LPS were abrogated. In conclusion, the OUA pre-treatment abolished the effect caused by LPS, suggesting that this finding may be related to the restoration of the interaction between FXYD2 and the studied membrane proteins.

## 1. Introduction

Inflammation, an indicator of an infection, is the initial response of the immune system following the invasion of a foreign particle [1]. Chronic and exacerbated neuroinflammation in the Central Nervous System (CNS) can have negative impacts on cells caused by the activation of glial cells, predominantly microglia, which release a significant amount of proinflammatory cytokines, such as IL-1 (Interleukin 1), IL-6 (Interleukin 6), TNF-α (tumour necrosis factor-alpha), and INF-γ (interferon-gamma) [2]. Among the various models of microglial activation, the use of lipopolysaccharide (LPS), found in the outer membrane of gram-negative bacteria, is widely utilized [3]. LPS activates the immune system and prompts the expression of nuclear factor κB (NF-κB) in the CNS [4], leading to an increase in proinflammatory cytokine gene expression [5].

The Na,K-ATPase is a crucial enzyme for the survival of cells and performs various functions such as preserving the cellular osmotic balance and controlling the membrane potential through ATP hydrolysis. This enzyme maintains high K^+^ and low Na^+^ concentrations inside the cells relative to the extracellular environment, thereby creating an optimal environment in the CNS for the release of neurotransmitters and efficient nerve transmission [6]. 

The Na,K-ATPase is composed of different isoforms of α (α1, α2, α3, and α4) and β (β1, β2, and β3) subunits and, in some cases, an additional subunit called FXYD [7]. The α1 isoform is expressed in all cell types, particularly in the kidney, which is a commonly studied tissue. The α2 isoform is found in cardiac and neuronal tissues, among others. The α3 isoform is present in neuronal tissues and ovaries, while the α4 isoform has only been described in sperm [8,9]. In neuronal tissues, the α1 and α3 isoforms are the main ones expressed, while both α1 and α2 are expressed in astrocytes and microglia [10,11,12]. The FXYD2 subunit has a role in the CNS and may act as an anchor molecule, helping the translocation of the glutamate transporter to the membrane [13,14].

In some disorders, such as epilepsy, hypoglycaemia, and ischaemia, Na,K-ATPase activity is decreased, thus impairing the Na^+^ gradient, which leads to neurotransmitter absorption, resulting in the blockade of reuptake and release of glutamate [15,16].

The text discusses the role of glutamate transporters, also referred to as Excitatory Amino Acid Transporters (EAATs), in the reuptake of the neurotransmitter glutamate [17]. There are five types of EAATs, with EAAT1 and EAAT2 being the primary carriers. These transporters are located in glial cells within the brain and are responsible for the reuptake of glutamate [18]. The transportation of glutamate occurs through an ion gradient created by Na,K-ATPase, and any alterations to the Na,K-ATPase can impact the glutamate transporter. EAAT1 is primarily expressed in the forebrain, while EAAT2 is primarily expressed in the cerebellar glia [19]. In LPS-induced neuroinflammation processes, a decrease in the EAAT2 and EAAT3 transporter mRNA levels can occur in the rat hippocampus, leading to glutamate-induced neurotoxicity [20]. Studies have reported that the expression of EAAT1/GLAST and glutamate uptake were significantly reduced in astrocyte cell cultures treated with LPS (10 ng/mL, 72 h) [21].

The research group has discovered that ouabain (OUA) can mitigate the adverse effects of LPS-induced neuroinflammation [22,23,24]. Ouabain is a type of cardiotonic steroid that originates from certain plants found in tropical and subtropical regions [25]. Cardiotonic steroids are known to obstruct the active transport of sodium and potassium ions through Na,K-ATPase [26].

Studies have demonstrated that ouabain (OUA) administered at nanomolar concentrations to rats can interact with Na,K-ATPase in the Central Nervous System (CNS) without inhibiting it, instead mediating cellular signalling processes, which results in the increased production of anti-inflammatory cytokines [16,27].

Although it is unclear if OUA can cross the blood-brain barrier or if its effects are due to its endogenous production in the CNS, OUA and other cardiotonic steroids have been demonstrated to function as neuroprotectors [28,29]. A recent discovery is OUA’s ability to modify the composition of the lipid membrane in the hippocampus and cerebellum [22,23,24]. In a previous study, OUA pre-treatment increased EAAT4 expression in the cerebellum [23].

The hypothesis of the study is that the administration of OUA will have an impact on the transport of glutamate in the hippocampus of rats that have been induced with LPS-induced neuroinflammation. The study aims to comprehend the relationship between OUA and the regulation of glutamate transport in the hippocampus and its potential impact on neuroinflammation. The hypothesis suggests that OUA may have a positive effect on the transport of glutamate and may potentially reduce the adverse effects of LPS-induced neuroinflammation.

## 2. Materials and Methods

### 2.1. Treatment of Animals with OUA and LPS

Four-month-old male Wistar rats (from the Biomedical Sciences Institute at the University of São Paulo) were housed under a 12 h light/dark cycle, with lights on at 7:00 a.m. The temperature was maintained at 25 °C, and the humidity was set at 50%, with free access to food and water. The animals were treated with saline, ouabain (OUA; from Sigma O3125, St. Louis, MO, USA), or Lipopolysaccharides (LPS; from Sigma L2630, St. Louis, MO, USA) between 9:00 a.m. and 11:00 a.m., following procedures approved by the College of Biomedical Animal Experimentation (COBEA). All procedures were approved by the Ethical Committee for Animal Research of the Biomedical Sciences Institute at the University of São Paulo, with approval code 77.

Wistar rats were distributed into four groups (*n* = 5 per group):

(1) CTR (control group): Animals received only an i.p. injection of saline solution and, after 20 min, another injection of saline solution (the second injection of saline is a control for the LPS).
(2) OUA-treated group: Animals received the first injection of OUA (i.p.) at a concentration of 1.8 µg/kg (concentration at nanomolar levels) and, after 20 min, an injection of saline solution (i.p.).
(3) LPS-treated group: Animals received an injection of saline solution (i.p.) first and, after 20 min, an injection of LPS at a concentration of 200 µg/kg (i.p.).
(4) OUA + LPS group: Animals received the first injection of OUA at a concentration of 1.8 µg/kg (i.p.) and, after 20 min, an injection of LPS at a concentration of 200 µg/kg (i.p.).

**Figure 1:**
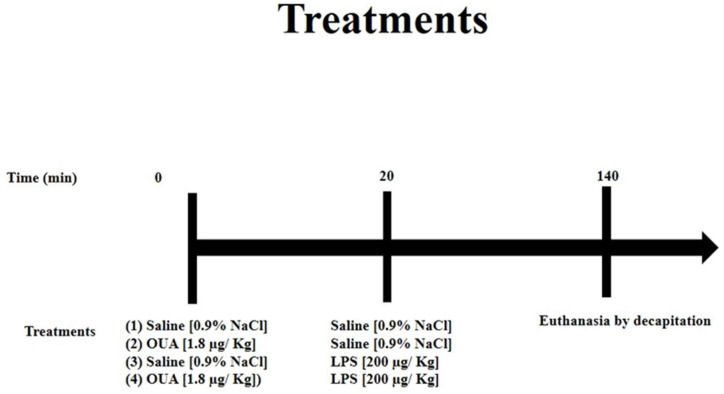
Diagrammatic representation of treatment chronology. All administration was performed via i.p. injection.

The time ranges and concentrations used for the treatment of the groups are shown in Figure 1. After 140 min of the initial injection, euthanasia by decapitation was performed, and the brains of each animal were rapidly removed and washed in ice-cold PBS solution. The hippocampus was dissected on a plate kept on ice and then immersed in liquid nitrogen and subsequently stored in a freezer at −80 °C until experiments were performed.

### 2.2. Preparation of Samples

Each sample was processed to prepare homogenates and a membrane fraction. Each hippocampus was initially homogenised in a mechanical potter in 5 mL of preparation buffer containing Tris-HCl 10 mM (Neon02643; pH 7.4), sucrose 0.32 M (Labsynth 01S2609.01.AG), EDTA 0.5 mM (Neon 01155) and MgCl_2_ 1 mM (Dinamica 1055-1000) and 10% protease inhibitor cocktail (AEBSF 2 mM, aprotinin 0.3 µM, bestatin 116 µM, E-64 1 µM, and leupeptin 1 µM; Sigma^®^ cod. P2714) to obtain the homogenate. Following homogenisation, 2 mL of the homogenate was removed for analysis of oxidative parameters. The remaining 3 mL was centrifuged for 20 min at 10,000× *g* at 4 °C, and then the supernatant was ultra-centrifuged at 100,000× *g* for 1 h at 4 °C. The pellet was resuspended in 500 µL of the preparation buffer to obtain the membrane fraction [30].

Total protein concentrations were determined using the method described by Bradford, using bovine serum albumin (BSA) as a standard [31]. 

### 2.3. Determination of Na,K-ATPase Activity

A membrane fraction protein (10 µg) was added to a reaction medium containing Tris-HCl 40 mM (pH 7.4), NaCl 120 mM (Dinamica 10.0294.078.00), KCl 20 mM (Labsynth, 01C1058.01.AH), MgCl_2_ 5 mM, and ATP 3 mM (Sigma^®^ cod. A26209) with or without OUA (10 µM or 1 mM) at 37 °C for 1 h. The reaction was then stopped by adding 100 μL of 1% phosphate-free SDS (Sigma, 436143). Subsequently, 100 µL of Fiske’s solution was added for the complexation of phosphate released from ATP hydrolysis [32]. The formation of the phosphomolybdate complex was determined spectrophotometrically at 660 nm.

In rodents, the α1 isoform of the Na,K-ATPase is 1000 times less sensitive to OUA than isoforms α2 or α3. The total activity of Na,K-ATPase was calculated by subtracting the values obtained from activities with and without 1 mM OUA (concentration required for inhibition of α1, α2, and α3 isoforms) [33]. 

The Na,K-ATPase α1 activity was calculated by subtracting the values obtained for the Na,K-ATPase activity with 10 µM OUA (the concentration required to inhibit α2 and α3 isoforms) from the activity with 1 mM OUA. The α2 and α3-Na,K-ATPase activity was calculated by subtracting the values obtained for Na,K-ATPase activity without OUA from the activity with 10 µM OUA. All experiments were performed in triplicate. Values are expressed as nmol Pi/min/mg of protein.

### 2.4. Determination of Glutamine Synthetase (GS) Activity

Hippocampal homogenate (100 µL) was added to a reaction medium (imidazole-HCl 100 mM, pH 7.2; glutamate 50 mM (Sigma, G1251); hydroxylamine-HCl 100 mM; MgCl_2_ 20 mM; and 2-mercaptoethanol 25 mM (Sigma 63689)) at 37 °C. The reaction was started by adding 10 mM ATP, and after 30 min, the reaction was stopped by adding 1.0 mL FeCl_3_ 370 mM/HCl 60 mM/TCA 3M [18]. The formation of gamma-glutamyl hydroxamate was spectrophotometrically determined at 540 nm. The activity of this enzyme was calculated from the molar extinction coefficient of gamma-glutamyl hydroxamate (0.7 × 10³ M/cm), and values were expressed in mM of gamma-glutamyl hydroxamate formed/min/mg protein.

### 2.5. Anti-FXYD2 Immunoprecipitation

For immunoprecipitation, 300 µg of membrane fraction protein was used. Samples were transferred to 500-µL conical tubes, where 2 µL of FXYD2 antibody (Santa Cruz^®^, sc-81876, San Diego, CA, USA) was added and vortexed at 15 min intervals for 2 h. Then, 20 µL of protein A/G plus agarose (Santa Cruz^®^, sc-2003, San Diego, CA, USA) was added and vortexed again at 15 min intervals for 2 h. In the intervals between the shakes, the samples were rested at 4 °C in a refrigerator. After 2 h, the tubes were centrifuged at 1000× *g* for 5 min at 4 °C. The supernatant was discarded, and the pellet was resuspended in 500 µL PBS, and the same procedure was performed three times. At the end of the last centrifugation, the pellet was resuspended in 48 µL of PBS and 12 µL of sample buffer (0.125 mM Tris-HCl (pH 6.8), 0.004% bromophenol blue, 10% 2-mercaptoethanol, glycerol 20%, and 4% SDS).

### 2.6. Western Blotting

The samples were electrophoresed on a gel. Each sample was diluted in ultrapure MilliQ water and sample buffer (0.125 mM Tris-HCl (pH 6.8), 0.004% bromophenol blue, 10% 2-mercaptoethanol, 20% glycerol, and 4% SDS). Subsequently, the samples were run on a 12.5% or 15% polyacrylamide gel for immunoprecipitation with FXYD2 (sodium dodecyl sulfate-polyacrylamide gel electrophoresis- SDS-PAGE; 1.5 M Tris running gel (pH = 8.8), 30.8%/2.7% acrylamide/bis-acrylamide, 10% SDS, 10% ammonium persulfate, and TEMED [N, N, N′, N′-tetramethylethylenediamine]; packing gel: Tris 0.5 M (pH = 6.8), 30.8%/2.7% acrylamide/bis-acrylamide, 10% SDS, 10% ammonium persulfate, and TEMED; running buffer: 25 mM Tris-Base, 192 mM glycine, and 0.1% SDS).

After running, the polyacrylamide gel proteins were transferred to a nitrocellulose membrane (UltraCruzTM) for 1 h (25 mM Tris-base transfer buffer, 192 mM glycine, and 20% methanol). Transfer efficiency was evaluated by staining the nitrocellulose membrane with Ponceau xylidine red solution (DYNAMICS). The membrane was blocked for 1 h with 5% BSA diluted in T-TBS (100 mM Tris-Base, 0.9% NaCl, and 0.1% Tween) to block the nonspecific binding of the antibodies.

Subsequently, the membrane was washed twice for 5 min each with T-TBS and incubated overnight with the primary antibodies (listed in Table 1); all antibodies have reactivity to the species used in the study, according to the suppliers’ datasheets. All antibodies were diluted in T-TBS. The following day, the membrane was washed three times for 10 min each and incubated for 1.5 h with the secondary antibodies (listed in Table 2).

The proteins recognised by the antibodies were detected using electrochemiluminescence. The L-Pix Cheni Molecular Imaging (Loccus^®^, San José, Brazil) photo documentation system and Image J software were used to quantify the immunoblots. The results are expressed relative to β-actin expression. Values are expressed in comparison to the control group.

### 2.7. Statistical Analysis

GraphPad Prism 5 software was used, and the values are expressed as the mean ± standard error of the mean (SEM). Data were analysed using one-way analysis of variance (ANOVA) followed by the Newman–Keuls post hoc test. The significance level was set at *p* < 0.05.

## 3. Results

### 3.1. OUA Attenuates the Decrease in α2 Isoform Expression in LPS-Treated Rat Hippocampus

Based on our previous studies [23,24,25], we investigated the potential mechanisms by which ouabain (OUA) could alleviate neuroinflammatory processes. To do so, we analysed the activity of the Na,K-ATPase first, as it has been reported that OUA can specifically interact with the α subunit of this pump [34]. 

**Figure 2:**
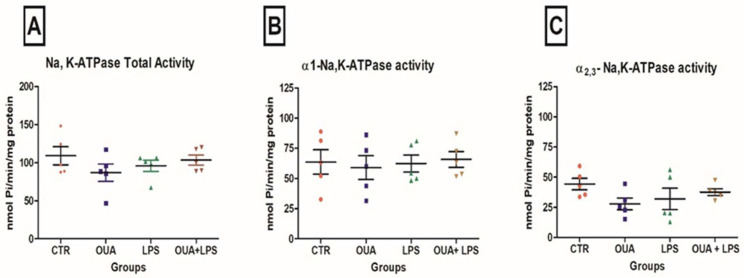
Na,K-ATPase activity in the hippocampus of rats treated with LPS and/or OUA. (**A**) Total Na,K-ATPase activity. (**B**) Activity of the α1 isoform of Na,K-ATPase. (**C**) Activity of the α2 and α3 isoforms of Na,K-ATPase. Red Circle—CTR group; Purple square—OUA group; Green Up Triangle—LPS Group; Orange downward triangle—OUA + LPS group. Data are presented as mean ± SEM (*n* = 5 per group) expressed as nmol/mg/min protein. Data were statistically evaluated using a one-way ANOVA followed by the Newman–Keuls post hoc test, and *p* < 0.05 was considered statistically significant.

Figure 2A shows no significant difference in total Na,K-ATPase activity in rats treated with OUA and/or LPS (F_(3,98)_ = 1.00674; *p* > 0.05). Likewise, as shown in Figure 2B, the OUA (58.78 ± 12.67 nmol Pi/min/mg protein), LPS (62.34 ± 7.06 nmol Pi/min/mg protein), and OUA + LPS groups (65.74 ± 6.55 nmol Pi/min/mg protein) did not differ from the control (F_(3,98)_ = 0.10816; *p* > 0.05). The same effect was observed for the activities of α2 and α3 isoforms of Na,K-ATPase (Figure 2C) (F_(3,98)_ = 1.5442; *p* > 0.05).

**Figure 3:**
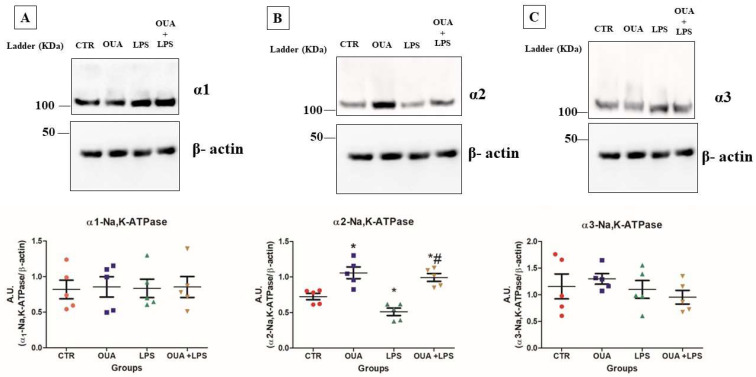
Western blot of the α isoform of Na,K-ATPase in the hippocampus of rats treated with LPS and/or OUA. (**A**) Western blot and densitometry analysis (arbitrary units of the α1/β-actin ratio). (**B**) Western blot and densitometry analysis (arbitrary units of α2/β-actin ratio). (**C**) Western blot and densitometry analysis (arbitrary units of α3/β-actin ratio). Red Circle-CTR group; Purple square-OUA group; Green Up Triangle-LPS Group; Orange downward triangle-OUA + LPS group. Data are presented as mean ± SEM, *n* = 5 per group. Data were statistically evaluated using a one-way ANOVA followed by the Newman–Keuls post hoc test, and *p* < 0.05 was considered statistically significant. * significantly different in comparison to control and # significantly different in comparison with LPS.

The expression of isoforms α1, α2, and α3 was also evaluated. None of the treatments altered the expression of α1 and α3 isoforms (Figure 3A,C) (F_(3,98)_ = 0.01547; *p* > 0.05 and F_(3,98)_ = 0.7427; *p* > 0.05). However, it was shown that OUA treatment increased α2 isoform expression (~20%; F_(3,98)_ = 17.48099; *p* < 0.05) in the hippocampal cell plasma membrane of these rats, whereas LPS decreased (~22%; F_(3,98)_ = 17.48099; *p* < 0.05) the expression of this isoform, and the OUA pre-treatment prevented this LPS effect (Figure 3B).

### 3.2. OUA Restores Levels of Glutamate Transporters (EAAT1 and EAAT2) in Rat Hippocampus Treated with LPS

**Figure 4:**
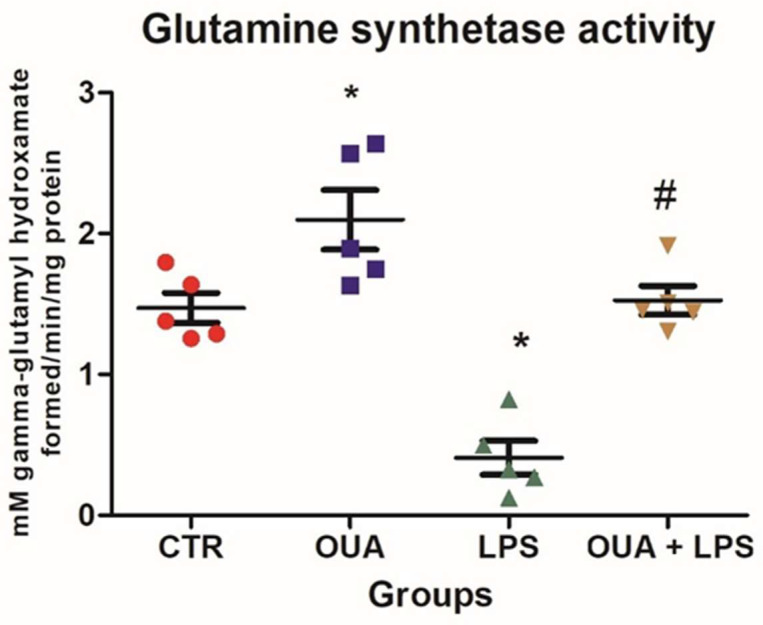
Glutamine synthase activity in the hippocampus of rats treated with LPS and/or OUA. Data are expressed as mean ± SEM of five animals. Red Circle-CTR group; Purple square-OUA group; Green Up Triangle-LPS Group; Orange downward triangle-OUA + LPS group. Results were analysed using a one-way analysis of variance (ANOVA) followed by the Newman–Keuls post hoc test, and *p* < 0.05 was considered statistically significant. * significantly different in comparison to control and # significantly different in comparison to the LPS group.

GS is an astrocyte-specific enzyme involved in LPS-induced inflammation [35]. LPS challenge is known to increase GS levels (0.74 ± 0.22 mM gamma-glutamyl hydroxamate formed/min/mg protein; F_(3,98)_ = 24.64; *p* < 0.05); therefore, its activity was also analysed. Figure 4 shows the inhibition of ~54% in GS activity in the LPS group, whereas the OUA group (1.96 ± 0.27 mM gamma-glutamyl hydroxamate formed/min/mg protein; F_(3,98)_ = 24.64; *p* < 0.05) showed an increase of ~25% in GS activity, as compared with the CTR group (1.59 ± 0.12 mM gamma-glutamyl hydroxamate formed/min/mg protein). In contrast, the OUA + LPS group (1.52 ± 0.10 mM gamma-glutamyl hydroxamate formed/min/mg protein) showed no change in GS activity compared to the CTR group (Figure 4). Notably, the group that received OUA pre-treatment demonstrated an attenuation of the effect induced by LPS.

Another marker for homeostasis is the glutamate transporter. These transporters move glutamate across the membrane, dependent on an ion gradient regulated by the Na,K-ATPase. EAAT1 and EAAT2 are located in astrocytes, and their main function is the reuptake of this neurotransmitter [36]. The expression levels of EAAT1 and EAAT2 in the membrane were analysed, taking these observations into account. It is also known that an increase in inflammatory cytokines, such as IL-1β and TNF, can suppress the expression of these glutamate transporters both in vitro and in vivo [37,38,39]. Therefore, we investigated the levels of these two important glutamate transporters in our study model.

**Figure 5:**
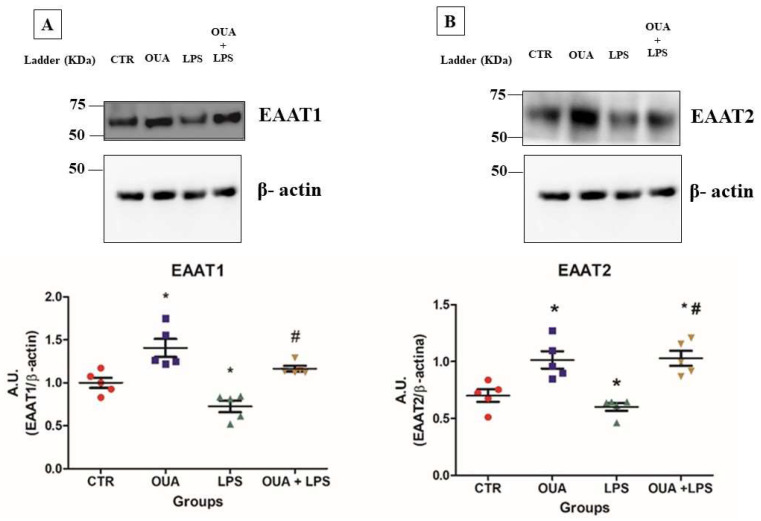
Western blotting of EAATs in the hippocampus of rats treated with LPS and/or OUA. (**A**) EAAT1 expression in rat hippocampal cell membrane. (**B**) EAAT2 expression in rat hippocampal cell membrane. Red Circle-CTR group; Purple square-OUA group; Green Up Triangle-LPS Group; Orange downward triangle-OUA + LPS group. Data are expressed as mean ± SEM (*n* = 5 per group). Results were analysed using a one-way analysis of variance (ANOVA) followed by the Newman–Keuls post hoc test, and *p* < 0.05 was considered statistically significant. * significantly different in comparison to control # significantly different in comparison to the LPS group.

Assessment of EAAT1 expression in the membrane showed that the LPS-treated group showed a significant reduction of approximately 22% in EAAT1 expression compared to the LPS group (F_(3,98)_ = 16.50; *p* < 0.05). The OUA group showed a 37% increase in EAAT1 expression compared to that in the CTR group (F_(3,98)_ = 16.50; *p* < 0.05). Again, it was demonstrated that OUA pre-treatment attenuated the effects of LPS (Figure 5A). A similar profile was observed for EAAT2, in which the LPS group showed a reduction of approximately 15% compared to the CTR group (F_(3,98)_ = 13.23; *p* < 0.05). The group treated with OUA showed an increase in the EAAT2 expression level by approximately 40% when compared with the CTR group. In addition, OUA pre-treatment attenuated the effects of LPS (Figure 5B) (F_(3,98)_ = 13.23; *p* < 0.05).

### 3.3. OUA Modulates the Interaction between FXYD2, α2 and Glutamate Transporters in Hippocampus of LPS-Treated Rats

An interesting correlation in our results is that the α2 expression profiles of Na,K-ATPase, EAAT1, and EAAT2 are similar. One hypothesis suggested by the expression profile of these proteins would be the presence of some common ligands that, by association, increased or decreased the expression of these proteins in the plasma membrane. There are few reports in the literature about this hypothesis, and Gegelashvili et al. (2007) have suggested that the FXYD2 subunit can be an anchor molecule because it is enriched with basic residues and may act in direct binding with negatively charged amino acids in the glutamate transporter C-terminal [13].

**Figure 6:**
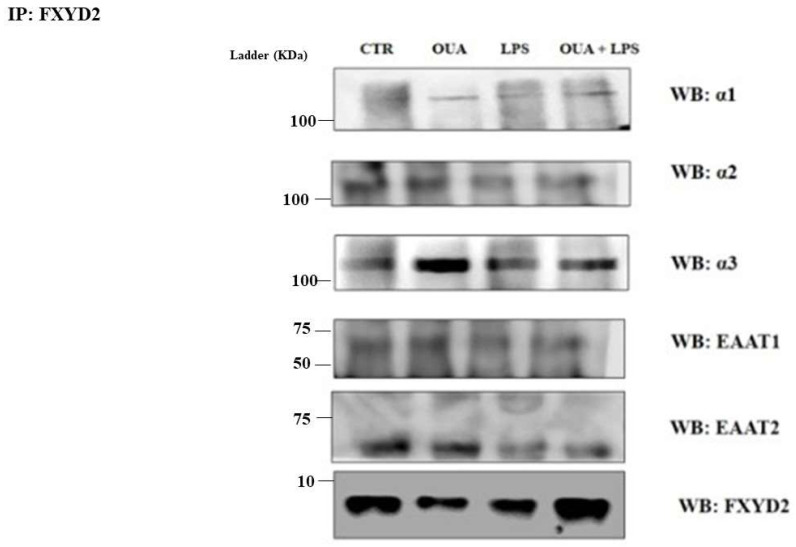
FXYD2 interaction with α1, α2, α3, EAAT1, and EAAT2 in the hippocampus of rats treated with LPS and/or OUA. Western blot (WB) for α1, α2, α3, EAAT1, and EAAT2 in rat hippocampal cell membrane fraction (*n* = 5).

Therefore, we analysed whether FXYD2 had any correlation with the effects of OUA administration on the protection process. To test this hypothesis, after immunoprecipitating FXYD2, we performed a Western blot assay for other proteins that could possibly interact with it. Figure 6 shows that OUA treatment also increased the interaction between FXYD2 and EAAT1, EAAT2, and between α2 and α3 subunits. LPS decreased all these interactions and may be responsible for the decreased EAAT1, EAAT2, and α2 expression. OUA pre-treatment restored these interactions, possibly returning these proteins to levels comparable to those in the membrane of the CTR.

## 4. Discussion

The hippocampus is a crucial area for neuroprotection research, as it is frequently affected by neurological diseases, such as Alzheimer’s, Parkinson’s, epilepsy, and stroke [40]. Due to the significant cognitive damage caused by these diseases, the hippocampus is a vital region for studying drugs that can counteract the harmful effects of neuroinflammation [41,42].

In this study, a treatment time of 140 min was chosen based on previous data that showed ouabain had anti-inflammatory and anti-apoptotic effects in the hippocampus when challenged with LPS-induced inflammation [27]. This effect is mediated by NF-κB modulation and changes in GFAP in the dentate gyrus, as well as the ability to suppress inflammation and maintain hippocampal BDNF levels [16,32,33]. Other studies have shown that ouabain can prevent oxidative stress and modulate lipid composition in neuronal regions [22,23,24]. Some studies suggest that low concentrations of ouabain can protect the heart and CNS against injury and inflammation [43,44], which is consistent with the concept of hormesis. Therefore, the study aims to explore the idea that ouabain could have a hormetic effect and challenge the CNS to obtain protection [45].

In addition, the attenuation effects of OUA against oxidative stress and the depletion of membrane lipids caused by LPS-induced neuroinflammation have also been reported [22,23,24]. Based on our previously published results, we sought to identify the possible mechanisms by which OUA could attenuate neuroinflammatory processes. For this reason, we analysed the activity of the Na,K-ATPase first.

No changes were found in the activity of total Na,K-ATPase, and their isoforms in any of the treatments studied (Figure 2). Studies have reported that treatment with LPS in rats for 4 h decreased the total Na,K-ATPase activity, and this change was related to a lower α2 and α3 isoform activity [46]. In another study, administration of LPS (2 mg/kg) caused a decrease in the total Na,K-ATPase activity, in this case, attributed to inhibition of the α1 isoform in the hippocampus [47]. It is noteworthy that in a study using rat cerebellum, no difference in the activity or expression of the Na,K-ATPase and its isoforms after treatment with LPS was found [23]. These results corroborate those found in rat cerebellum. However, it has been shown that when OUA was administered intracerebroventricularly (icv), there was an increase in α3 isoform expression in the rat frontal cortex, which led to an increase in locomotor activity in animals [48].

In a previous study, the treatment of astrocytes in culture with nanomolar and picomolar concentrations of OUA for 4 h was shown to counteract the increase in α-subunit expression caused by LPS [45]. It is noteworthy that the administration of OUA alone resulted in an increase in the expression of the α2 isoform (as depicted in Figure 3B). The treatment with OUA elevated the expression of the α2 isoform, which is primarily present in glial cells, particularly astrocytes [13]. In contrast, LPS caused a decrease in its expression. This isoform plays a crucial role in the CNS, as it regulates glycolysis and mitochondrial activity due to the high ATP consumption by the Na,K-ATPase in the brain [46]. Additionally, the α2 isoform has a significant role in regulating the central effects of LPS, with implications for associated neuroinflammatory processes [34].

Based on the results obtained for Na,K-ATPase, it is hypothesized that the effects of OUA pre-treatment do not impact the activity of this enzyme. However, as OUA is recognized as a specific ligand for Na,K-ATPase, it is speculated that OUA may bind to this enzyme and act as a cellular receptor, triggering a signalling cascade and mitigating the effects of LPS.

The study suggests that cardiac glycosides may interact with a group of transcription factors, which regulate various cell and organism functions through low-weight molecular ligands, such as hormones. This could be another possible explanation for the action of OUA, but further studies are needed for confirmation.

The study hypothesized that OUA’s effect on the α2 isoform expression in LPS-treated rat hippocampus might lead to changes in glutamate transporters EAAT1 and EAAT2, as these are related to the α2 isoform. The study further investigated the activity of GS as it is known to modulate inflammation processes and glutamate transport homeostasis [49,50].

Sharma et al. (2016) showed that hippocampal astrocytes from LPS-treated rats (50 µg/kg via icv) demonstrated a reduction in GS expression, an effect that the authors correlated with an increase in the number of apoptotic astrocytes [51]. LPS treatment of 3T3-L1 cells (murine fibroblasts) caused a decrease in GS expression [50]. Decreased GS activity has also been reported in patients with Alzheimer’s disease [52].

GS activity is an element that correlates glutamate homeostasis with inflammation [50]. Evidence has shown that GS is highly sensitive to oxidative processes [53,54], which is consistent with the oxidative stress damage found in a previous study [24].

It is important to report that OUA pre-treatment was able to counteract the effects of LPS on this important enzyme normalising its role in glutamate homeostasis, possibly because OUA decreased the oxidative effects of LPS. 

EAATs are responsible for extracellular glutamate homeostasis and are highly expressed in glial cells, and their altered expression or function is observed in neuropathologies [17,55]. In the present study, we demonstrated a significant modulation in the expression of EAAT1 and EAAT2, and we verified that OUA alone could increase the expression of both transporters after treatment with LPS, causing a decrease in their expression. In contrast, pre-treatment with OUA blocked the effect of LPS, suggesting a possible neuroprotective effect of this compound (Figure 5).

Studies suggest that there is a close relationship between the dysregulation of glutamate homeostasis and neuroinflammatory processes. An imbalance of both neurotransmitter release and uptake may potentiate deleterious effects such as oxidative stress, mitochondrial dysfunction, increased inflammatory cytokines, and excitability. Studies have reported that LPS-treated astrocyte cell culture (10 ng/mL, 72 h) showed decreased EAAT1/GLAST expression and significantly decreased glutamate uptake [21]. This effect was also observed in foetuses of Sprague-Dawley rats treated with LPS (200 μg/kg i.p.) [56]. In addition, we demonstrated a neuroprotective effect of OUA against neurotoxicity induced by 4 h excitotoxic insults in the rat cortical neurons [57].

Therefore, the present data reinforce that in our in vivo study model, OUA anti-inflammatory action is linked to a reversal of LPS-induced decrease of EAAT1 and EAAT2 expression in the hippocampus.

In a previous study, we showed that OUA pre-treatment caused EAAT4 to increase in the cerebellum [23], demonstrating the ability of OUA to modulate the expression of glutamate transporters. Rose et al. (2009) showed a dual effect of OUA administration on astrocytes, where concentrations in the 1 µM OUA range caused glutamate transport activation, whereas higher ranges inhibited glutamate transport [58]. Thus, they demonstrated that low concentrations of OUA can affect this transport, improving glutamate uptake capacity and decreasing neurotoxicity. The effects of OUA on these transporters may be explained by evidence that both GLAST/EAAT1 and GLT-1/EAAT2 interact with the Na,K-ATPase [58,59], causing a change in the activity or structure of the enzyme, which has a direct effect on these transporters.

Rose and colleagues suggested that glutamate transporters and Na,K-ATPase form a multiprotein complex, although their components have not yet been reported. They also reported that any conformational change in this complex could alter glutamate transport [58]. Therefore, our results showed that FXYD2 expression might be correlated with the attenuating effects of OUA when challenged with LPS (Figure 6). In addition, OUA treatment alone enhanced this interaction, indicating an explanation for the increased levels of membrane glutamate transporters (Figure 5). However, more experiments have to be performed to confirm this hypothesis.

**Figure 7:**
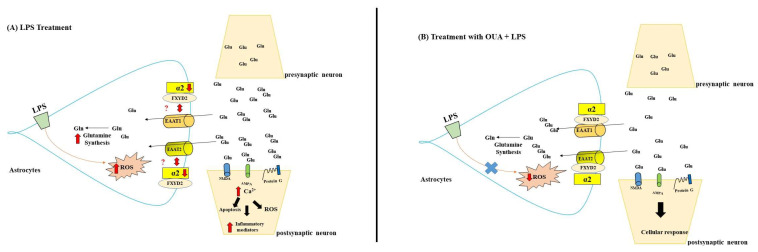
Scheme summarising the main modulated effects of OUA and LPS on rat hippocampus. (**A**) Effects caused by LPS. (**B**) Effects caused by OUA treatment after LPS-induced neuroinflammation in rats. Glu, glutamate; LPS, lipopolysaccharide; OUA, ouabain; ER, endoplasmic reticulum; GS, glutamine synthetase; SERCA, calcium ATPase sarcoplasmic reticulum membrane; NKA, Na,K-ATPase; Ca^2+^, calcium; ROS, reactive oxygen species; and TLR4, toll-like receptor 4.

Based on our previous results of studies on oxidative stress [24], we suggest that the increase of ROS by LPS may cause oxidation of the basic amino acids responsible for the interaction with glutamate transporters, thus decreasing this interaction (Figure 7A). OUA treatment attenuated oxidative stress and enabled the restoration of this interaction (Figure 7B). However, further studies are needed to verify this hypothesis.

## 5. Conclusions

Our results demonstrate that pre-treatment with a low dose of OUA can reduce the impact of LPS. LPS administration causes changes, including reduced expression of the α2 isoform of Na,K-ATPase, as well as EAAT1 and EAAT2 glutamate transporters, leading to increased excitotoxicity as evidenced by elevated glutamate levels in the synaptic cleft.

It is worth mentioning that the decreased expression of the α2 isoform of Na,K-ATPase, EAAT1, and EAAT2 in the membrane by LPS treatment may contribute to FXYD2 expression, as LPS reduces the interaction between this proteolipid and these proteins in the membranes.

In contrast, OUA alone resulted in a stronger interaction between FXYD2 and the α2 isoform of Na,K-ATPase, as well as between EAAT1 and EAAT2. OUA pre-treatment thus restored the lost interactions. It is also noteworthy that OUA pre-treatment eliminated the effect caused by LPS, suggesting that this may be related to the restoration of the interaction between FXYD2 and the studied membrane proteins.

It is noteworthy that this pioneering study suggests that OUA, a classic cardiotonic steroid, may modulate the interaction between FXYD2 and glutamate transporters. However, further experiments are required to confirm this hypothesis. This novel finding provides new avenues for exploring the neuroprotective action of cardiotonic steroids and offers insights into their potential use in the treatment of neuroinflammation.

## Figures and Tables

**Table 1:** Primary Antibodies.

Antibody	Code	Dilution for Membrane Fraction	Dilution for Immunoprecipitation	Molecular Weight (KDa)
FXYD2 (Mouse)	sc-81876	-	1:250	7
β-actin (Mouse)	sc-81178	1:500	-	45
α1 (Mouse)	sc-21712	1:500	1:250	110
α2 (Rabbit)	CSB-PA020068	1:500	1:200	110
α3 (Mouse)	MA3-915	1:1000	1:250	110
EAAT1 (Rabbit)	sc-515839	1:500	1:200	65
EAAT2 (Rabbit)	sc-365634	1:500	1:200	65

**Table 2:** Secondary Antibodies.

Antibody	Code	Dilution for Membrane Fraction	Dilution for Immunoprecipitation
Mouse	sc-516102	1:2000	1:1000
Rabbit	sc-2357	1:7000	1:2000

## Data Availability

The datasets generated and analyzed during the current study are available from the corresponding author on reasonable request.
